# Clinical utility and reproducibility of intravoxel incoherent motion MRI in rectal cancer: a structured narrative review

**DOI:** 10.3389/fmed.2026.1925061

**Published:** 2026-10-01

**Authors:** Ali S. Alyami, Meaad Elbashir, Sara Ali, Turkey Refaee, Wael Ageeli, Bandar Alwadani, Naif A. Majrashi, Abdullah A. Althobity

**Affiliations:** 1 Department of Diagnostic Radiography Technology, College of Nursing and Health Sciences, Jazan University, Jazan, Saudi Arabia; 2 Department of Radiological Sciences and Medical Imaging, College of Applied Medical Sciences, Majmaah University, Majmaah, Saudi Arabia

**Keywords:** rectal cancer, intravoxel incoherent motion, diffusion-weighted imaging, magnetic resonance imaging, reproducibility, nodal staging, treatment response, radiomics

## Abstract

**Introduction:**

Magnetic resonance imaging (MRI) is a crucial imaging modality for the management of rectal cancer. Conventional MRI provides detailed anatomical information but has a limited capacity to evaluate tumor biology. Intravoxel Incoherent Motion (IVIM) is an advanced functional MRI technique that simultaneously measures tissue cellularity and microvascular perfusion without contrast agents, providing deeper insights into the tumor microenvironment. This narrative review critically examines the current evidence on the clinical usefulness of IVIM MRI in rectal cancer, focusing on three key areas: tumor characterization and staging, prediction and monitoring of treatment response, and the reproducibility and limitations of IVIM parameters.

**Methods:**

A literature search was conducted in vid MEDLINE, PubMed, Embase, Web of Science Core Collection, and Google Scholar from 2015 to October 2025. The search included keywords such as “intravoxel incoherent motion OR IVIM” and “rectal cancer OR colorectal cancer.” Original studies with at least 10 patients were included; reviews, case reports, and animal studies were excluded.

**Results:**

The true diffusion coefficient D appears to be the most biologically interpretable and reproducible IVIM-derived parameter, with several studies linking lower D values to greater cellularity or more aggressive tumor features; however, findings are not uniform across all cohorts. While the perfusion fraction (f) has shown associations with markers of aggressiveness in some studies, its clinical utility is limited by poor reproducibility. For nodal staging, primary-tumor IVIM histogram models have shown promising diagnostic performance, although most evidence remains from single centers and is insufficiently validated. When assessing the response to neoadjuvant chemoradiotherapy (nCRT), a post-treatment increase in D appears to be the most consistent IVIM-derived marker of treatment response. However, its clinical use still requires standardized acquisition and external validation while perfusion parameters (f and D*) exhibit poor test-retest reproducibility. Combining IVIM with complementary techniques, such as diffusion kurtosis imaging or radiomics, may improve predictive performance compared with individual parameters.

**Conclusion:**

IVIM-DWI provides non-contrast quantitative information on diffusion and perfusion in rectal cancer. Current evidence suggests that D is the most reproducible and biologically interpretable IVIM parameter, whereas f and D* remain limited by poor repeatability and protocol dependence. IVIM may add value for tumor characterization, nodal risk assessment, and treatment-response evaluation, particularly when combined with histogram analysis, DKI, or radiomics; however, standardized acquisition protocols and external validation are required before routine clinical implementation.

## 1 Introduction

Colorectal cancer remains a major global health burden. According to GLOBOCAN 2022, more than 1.9 million new colorectal cancer cases and approximately 904,000 deaths occurred worldwide, making colorectal cancer the third most commonly diagnosed malignancy and the second leading cause of cancer-related mortality globally (1). Its incidence is influenced by a combination of genetic, socioeconomic, and lifestyle factors, including inherited syndromes such as familial adenomatous polyposis and Lynch syndrome. The rising burden of rectal cancer has been attributed partly to population aging and lifestyle-related risk factors. A notable demographic shift has also been observed in rectal cancer epidemiology. Incidence and mortality are declining among individuals aged 50 and older, primarily due to enhanced screening protocols and early detection. In contrast, younger adults have shown a persistent annual increase in incidence of approximately 1.5%. This age group is often diagnosed at a more advanced stage, and early-onset cases are forecast to represent approximately 23% of all rectal cancer diagnoses by the year 2030 (2). Prognosis is determined by the presence of distant metastases, mesorectal fascia invasion, lymph node spread, tumor mural (T1–T2) and extramural (T3–T4) involvement, and dissemination. The diagnosis and treatment planning of rectal cancer remain clinically challenging. Early-stage disease may present with nonspecific symptoms, which can delay diagnosis. Consequently, a substantial proportion of patients present with advanced-stage disease, having missed the optimal window for curative-intent treatment (3, 4).

Magnetic resonance imaging (MRI) has become a fundamental modality for assessing rectal cancer, providing essential insights into its morphology and potential biological behavior (5). It provides detailed anatomical and functional information for evaluating the rectal wall, lateral pelvic sidewall, and mesorectum. For example, MRI defines tumor location within the rectum or anal canal and its relationship to key anatomical landmarks and critical anatomical structures (such as the sphincter complex, anorectal junction, anterior peritoneal reflection, sigmoid take-off, and mesorectal fascia (5). The standard rectal MRI protocol uses high-resolution T2-weighted imaging to precisely evaluate the extent of tumor infiltration (6) and diffusion-weighted imaging (DWI) to differentiate tumor from non-tumor tissue. However, conventional sequences may have limitations in detecting extramural vascular invasion (EMVI), malignant nodal disease, early-stage tumors, and minimal residual disease after treatment (7).

Advanced quantitative MRI techniques, such as intravoxel incoherent motion (IVIM) and diffusion kurtosis imaging (DKI), provide biomarkers that may characterize tumor cellularity, perfusion, heterogeneity, and treatment-related microstructural change. IVIM provides information on microvascular perfusion and water diffusion in living tissues, whereas DKI characterizes non-Gaussian water diffusion and may also provide insights into tissue heterogeneity and microstructural complexity. These sequences provide valuable quantitative data on tumor characteristics and have been widely used to assess various cancers, including prostate cancer (7).

The clinical applications of these sequences in rectal cancer include tumor staging, prediction of lymph node involvement, prognostic assessment, and evaluation of response to neoadjuvant therapy (NAT) regimens (7–10). Although there is a growing number of studies exploring IVIM in rectal cancer, the findings remain inconsistent and sometimes conflicting. Variations in acquisition protocols, b-value choices, region-of-interest analysis, and fitting algorithms have led to significant differences across studies. As a result, the clinical significance of IVIM parameters, especially the perfusion-related ones (f and D*), is still debated. This review aims to thoroughly analyze the current literature on IVIM in rectal cancer, emphasizing key areas such as tumor characterization, prediction of treatment response, and the reproducibility and methodological challenges that impact the clinical application of IVIM-derived imaging biomarkers.

Previous reviews have discussed the application of advanced diffusion MRI, including IVIM, in oncology, including rectal cancer characterization, nodal assessment, and evaluation of treatment response (11–14). However, important challenges affecting the clinical translation of IVIM remain insufficiently resolved, particularly the reproducibility of IVIM-derived parameters, instability of perfusion-related metrics, methodological heterogeneity in acquisition and post-processing, and limited external validation of proposed imaging biomarkers. Therefore, the present review builds on the existing literature by critically synthesizing IVIM evidence according to biological interpretability, reproducibility, methodological robustness, and translational readiness, while also considering emerging histogram-based and radiomics approaches.

## 2 Technical principle of IVIM

Several diffusion-weighted MRI techniques have been investigated in rectal cancer (Table 1). The basic principles of IVIM have been described previously. Essentially, DWI quantifies signal loss resulting from the Brownian motion of water molecules in tissues when diffusion-sensitizing gradients are applied. In 1986, Le Bihan et al. introduced DWI based on IVIM (15). IVIM is a type of DWI that uses multiple b-values (which indicate the strength of diffusion gradients) to distinguish between microvascular perfusion and true molecular water diffusion within tissue. Unlike standard DWI, IVIM does not need a contrast agent. The IVIM-DWI model yields the diffusion coefficient (D) and the perfusion-related coefficients (D* and f). Specifically, f indicates the volume share of microcirculation in the overall signal, D* reflects random motion of microvascular blood, and D represents diffusion of pure water molecules. The signal decay can be modeled with a biexponential function of b-values.

$$S(b)/S_{0}=fe^{-b(D+D*)}+(1-f)e^{-bD}$$

Where, S (b): signal intensity at a given b-value, S0: signal intensity at b = 0,D: true diffusion coefficient, D*: pseudo-diffusion coefficient and f: perfusion fraction.

**Table 1:** Diffusion-weighted MRI techniques used in rectal cancer.

DWI method	Core principle	Parameters	Investigational applications
Mono-exponential DWI (13, 21)	Standard model using at least two b-values to quantify water diffusion.	ADC (Apparent Diffusion Coefficient)	Tumor detection, characterization and localization. Differentiating fibrosis from residual tumor post-treatment. General assessment of treatment response.
Intravoxel Incoherent Motion (IVIM) (13, 22)	Biexponential model that separates true molecular diffusion from microcapillary perfusion.	D (True Diffusion Coefficient) D* (Pseudo-diffusion Coefficient) f (Perfusion Fraction)	Assessing and predicting treatment response (e.g., pathological complete response (pCR) prediction. Correlation with tumor grade and histological features. Improving nodal staging.
Diffusion Kurtosis Imaging (DKI) (13, 23)	Non-Gaussian model that quantifies the heterogeneity of the microcellular environment.	MK (Mean Kurtosis) MD (Mean Diffusivity)	Assessment of tissue cellularity, heterogeneity, and microstructural complexity. Differentiating tumor stage and grade. Evaluating response to neoadjuvant therapy.

Using the biexponential model, IVIM parameters (D, D*, and f) can be estimated as shown in Figure 1 (16). In the biexponential IVIM model, the perfusion component (f and D*) is linked to the rapid signal decay at low b-values. Conversely, the true diffusion component (D) corresponds to the slower signal decay at high b-values. Despite its physiological appeal, the biexponential IVIM model has significant technical limitations. Parameter estimation requires nonlinear fitting of multiple signal components and is therefore sensitive to image noise and low signal-to-noise ratio (SNR). This problem particularly affects the perfusion-related parameters f and D*, which are predominantly determined by the rapid signal decay occurring at low b-values. Consequently, motion, insufficient sampling of low b-values, or a suboptimal distribution of b-values can substantially alter their estimates (17). D* is especially unstable because its estimation depends on the steep early portion of the signal-decay curve and may vary considerably according to SNR and the fitting algorithm. The fitting strategy can influence IVIM parameter estimation in rectal cancer. Fusco et al. (18) compared multiple IVIM fitting approaches in 40 patients with locally advanced rectal cancer and reported that a modified variable-projection algorithm produced a better model fit than conventional nonlinear least-squares and segmented methods. These results further indicate that IVIM estimates are influenced by both acquisition parameters and the post-processing method used for parameter derivation. In addition, covariance between f and D* can further destabilize biexponential fitting. These technical limitations provide an important explanation for the substantially poorer reproducibility of f and D* compared with D and should be considered when interpreting perfusion-related IVIM findings (19, 20).

**Figure 1:**
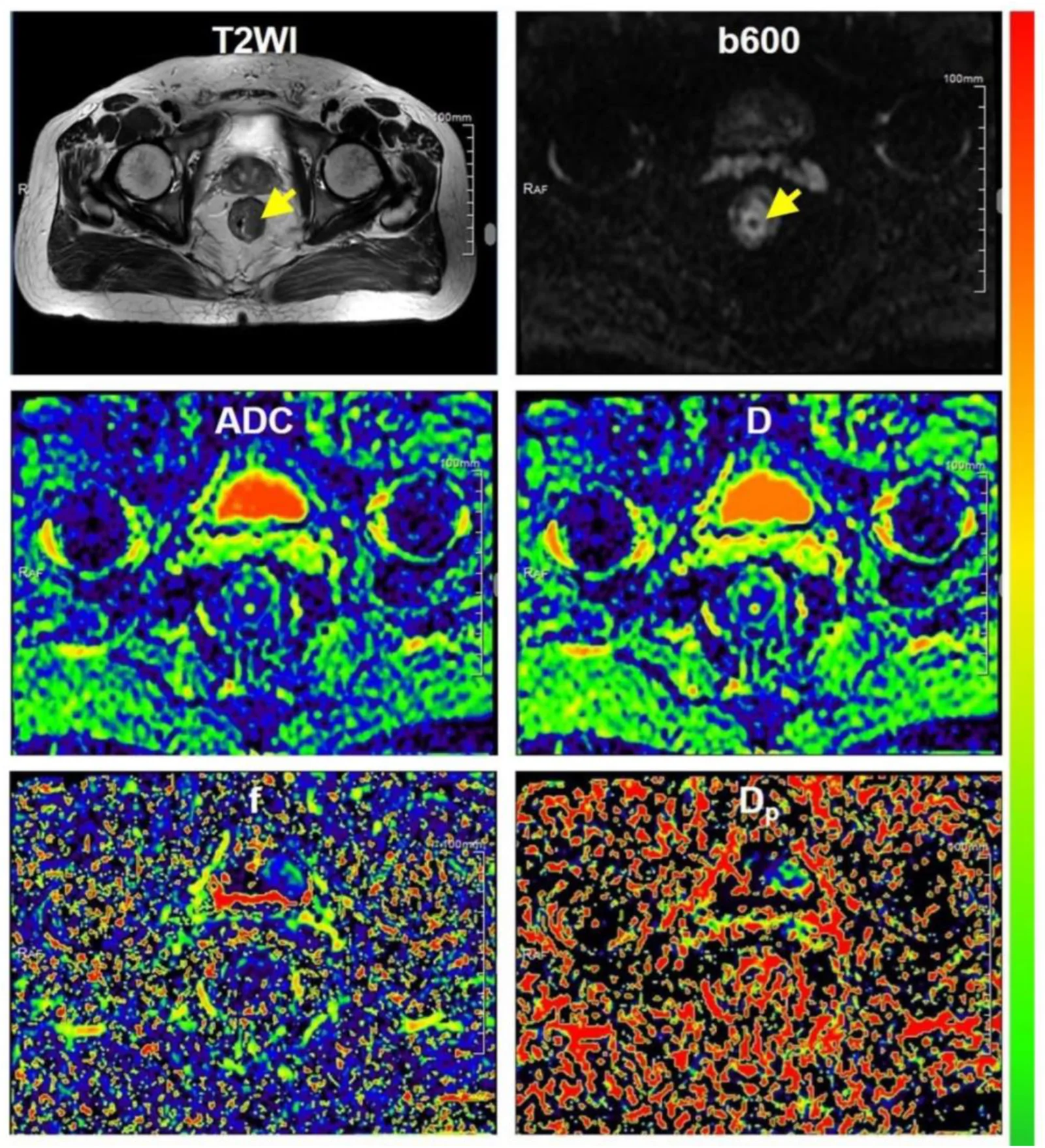
Representative MR images of a patient with high-grade rectal cancer. The yellow arrows indicate the tumors. The top row shows T2-weighted and DWI obtained at b = 600 s/mm^2^. The middle row shows ADC and D maps, and the bottom row shows f and D* maps. Yellow arrows indicate the tumor. All parametric maps use a consistent color scale from blue to red, representing the minimum and maximum values, respectively. The displayed ranges are 0.0–5.0 × 10^−3^ mm^2^/s for ADC and D, and 0.0–80.0 × 10^−3^ mm^2^/s for D*. Adapted with permission from Figure 3 “Representative MR images of a patient with high grade rectal cancer.” in (16), licensed under CC BY 4.0.

## 3 Literature search strategy and study selection

### 3.1 Literature search strategy

This narrative review was conducted using a structured literature search to enhance transparency and reproducibility. Electronic databases, including Ovid MEDLINE, PubMed, Embase, Web of Science Core Collection, and Google Scholar were searched for studies published between January 2015 and October 2025. PubMed was searched in addition to Ovid MEDLINE to identify recently indexed and ahead-of-print records and to cross-check retrieval, while Web of Science was included to broaden multidisciplinary and citation-based coverage. The search strategy included combinations of the following keywords: (“intravoxel incoherent motion” OR IVIM) AND (rectal cancer OR colorectal cancer OR LARC OR locally advanced rectal cancer OR rectal neoplasm OR rectal adenocarcinoma) AND (MRI OR magnetic resonance imaging).

### 3.2 Study selection

Inclusion criteria were original studies that investigated IVIM in rectal cancer with at least 10 patients. Studies published in English that used IVIM techniques in rectal cancer and were published from January 2015 through October 2025 were included. Editorials, case reports, animal studies, and review articles were excluded. The following data were extracted from each study: study design, sample size, imaging protocol, IVIM parameters evaluated (D, D*, and f), and the main clinical findings. Because this was a structured narrative review, no meta-analysis or formal risk-of-bias assessment was performed; however, study design, sample size, validation status, and methodological limitations were considered when interpreting the evidence. While duplicate screening was not performed, efforts were made to minimize bias through structured data collection and consistent application of eligibility criteria. This review was designed as a structured narrative review rather than a systematic review or meta-analysis. The purpose was to provide a critical synthesis of the current evidence on IVIM MRI in rectal cancer, with emphasis on clinical applications, reproducibility, methodological heterogeneity, and barriers to clinical translation. Therefore, PRISMA-based screening, duplicate independent review, and formal risk-of-bias assessment were not performed. However, to improve transparency, we used predefined eligibility criteria, extracted key study characteristics, and interpreted the findings in light of study design, sample size, validation status, and methodological limitations.

## 4 Application of IVIM in rectal cancer

Several studies have investigated IVIM for rectal cancer staging (24, 25) and the evaluation of treatment response. Table 2 shows the summaries of these studies. IVIM may help differentiate metastatic from nonmetastatic mesorectal lymph nodes, although reported diagnostic performance varies across studies. Previous studies found that metastatic lymph nodes showed significantly lower D and D* values than nonmetastatic lymph nodes (24, 25).

**Table 2:** Representative IVIM studies in rectal cancer according to clinical application.

Clinical application	Study/year	Study design/sample size	Main parameters/method	Main findings	Interpretation/limitation
Reproducibility	Sun et al., 2017 (19)	Prospective; *n* = 26	ADC, D, f, D*; test–retest IVIM MRI	ADC and D showed better repeatability than f and D*. Test–retest repeatability coefficients were 19.1% for ADC, 24.5% for D, 126.3% for f, and 197.4% for D*.	Demonstrates that D is more reliable than perfusion-related parameters. f and D* should be interpreted cautiously in longitudinal assessment.
Reproducibility/correlation with DCE-MRI	Yang et al., 2019 (20)	Prospective; *n* = 47	IVIM and DCE-MRI; f, D*, fD*, Ktrans, Kep	Moderate correlations were observed between f and DCE-derived parameters. Reproducibility was moderate for f and lower for D*.	Supports partial biological overlap between IVIM perfusion parameters and DCE-MRI, but reproducibility remains limited.
Perfusion correlation/biological validation	Bakke et al., 2019 (30)	Prospective; *n* = 94	IVIM, DCE-MRI, DSC-MRI	IVIM perfusion-related parameters showed limited and inconsistent correlation with DCE/DSC perfusion markers. No clear correlation was found between IVIM parameters and microvessel density.	Suggests that IVIM perfusion parameters do not directly replace DCE/DSC perfusion biomarkers.
Tumor differentiation	Li et al., 2021 (31)	Retrospective; *n* = 98	IVIM and DCE-MRI	f was higher in poorly differentiated tumors and showed moderate diagnostic performance for tumor grade. D and fD showed no significant difference.	f may reflect tumor vascularity or aggressiveness, but performance was moderate and less robust than DCE-MRI parameters.
Histopathological correlation	Surov et al., 2017 (22)	Prospective preliminary study; *n* = 17	D, f, D* correlated with histology	f correlated with vessel count and vessel area; D showed a trend toward inverse correlation with cellularity and Ki-67.	Provides biological validation, but sample size was small.
Histogram histopathological correlation	Meyer et al., 2021 (37)	Retrospective; *n* = 17	IVIM histogram parameters; cellularity, Ki-67, MVD	f histogram parameters correlated with microvessel density, while ADC and f entropy correlated with Ki-67. No significant correlation was found with tumor cellularity.	Suggests IVIM histogram analysis may capture vascularity and proliferation, but evidence remains exploratory.
Correlation with DCE-MRI	Sun et al., 2019 (55)	Retrospective; *n* = 97	IVIM and DCE-MRI parameters	Moderate correlations were observed between selected IVIM perfusion parameters and DCE-MRI parameters.	Indicates partial overlap between IVIM and DCE perfusion information, but correlations were not strong enough to establish interchangeability.
Tumor stage/clinicopathological features	Sun et al., 2022 (35)	Retrospective; *n* = 82	IVIM, ADC, DCE-MRI, texture analysis	IVIM parameters did not significantly differentiate pT1–2 from pT3a tumors.	Highlights inconsistent performance of IVIM for early T-stage discrimination.
Clinicopathological correlation	Xu et al., 2024 (56)	Prospective; *n* = 42	IVIM, DCE-MRI, R2*	D was negatively correlated with imaging T and N stages. D* was positively correlated with T stage and tumor markers. f was negatively correlated with T stage and R2*.	Suggests IVIM parameters may reflect tumor burden and hypoxia-related features, but validation is needed.
Risk stratification	Chai et al., 2025 (38)	Retrospective; *n* = 75	IVIM and DCE-MRI	f and D* increased across higher risk groups. Combined D* + f + Kep (DCE-MRI), showed improved diagnostic performance.	Supports multiparametric modeling rather than isolated IVIM interpretation.
Tumor grading	Geng et al., 2020 (8)	Prospective; *n* = 58	IVIM and DKI	All parameters except D* differentiated low-grade from high-grade tumors. Combined D, f, and Kapp achieved high diagnostic performance.	Combining IVIM with DKI improves tumor grading by capturing cellularity, perfusion, and heterogeneity.
KRAS mutation prediction	Xu et al., 2018 (39)	Retrospective; *n* = 51	IVIM and ADC	KRAS-mutant tumors had lower D and higher D*. f was not significantly different.	Suggests IVIM may help predict molecular status, but evidence remains preliminary.
Tumor grade/prognostic markers	Sun et al., 2018 (36)	Prospective single-center; *n* = 52	ADC, D, f, D*	f, D*, and ADC differed among tumor differentiation groups. D showed a decreasing trend with poorer differentiation but was not statistically significant.	Perfusion-related parameters may reflect tumor differentiation, although findings require validation.
Clinicopathological factors/PDC grade	Zhou et al., 2023 (10)	Prospective single-center; *n* = 51	D, D*, f, ADC	D, D*, and ADC were higher in pT1–2 than pT3–4 tumors. f correlated strongly with poorly differentiated clusters and showed high diagnostic accuracy.	Suggests IVIM may reflect tumor aggressiveness, especially when linked to pathological grading systems.
Pathological prognostic factors	Zhou et al., 2025 (33)	Prospective single-center; *n* = 162	DWI, IVIM, DKI	Higher f was independently associated with lymph node metastasis and EMVI. DKI parameters, especially MK, showed stronger performance for poor prognostic factors.	Indicates that IVIM may add value, but DKI may outperform IVIM for some prognostic assessments.
Synchronous distant metastasis (SDM)	Ding et al., 2022 (57)	Retrospective multicenter; *n* = 169	IVIM-DKI histogram parameters and clinical variables	The SDM group showed a lower 90th-percentile f value than the non-SDM group (29.4 ± 8.4% vs. 35.0 ± 17.8%, *p* = 0.005).	Supports the value of combined imaging-clinical models rather than IVIM alone.
				A combined model including f, tumor location, and CA19-9 achieved AUC = 0.801.	
Tumor budding/prognostic stratification	Yang et al., 2025 (41)	Retrospective single-center; *n* = 189	IVIM-derived histogram analysis	Histogram-derived D percentiles predicted high-grade tumor budding and showed good performance in training and validation cohorts.	Suggests D histogram metrics may capture aggressive tumor biology and prognosis.
FOCUS-IVIM tumor grading	Cheng et al., 2021 (58)	Retrospective single-center; *n* = 88	FOCUS-IVIM at 3T; ADC, D, D*, f	Tumor f and normalized IVIM parameters correlated with histological grade. f showed strong diagnostic performance.	High-resolution FOCUS-IVIM may improve image quality and grading performance, but external validation is required.
Nodal staging	Yu et al., 2016 (24)	Prospective study; *n* = 52	IVIM assessment of mesorectal lymph nodes	The metastatic group had significantly lower D and D* values than the nonmetastatic group (*P* < 0.01). IVIM helped differentiate metastatic from nonmetastatic mesorectal lymph nodes.	Suggests potential value for nodal characterization, but direct lymph-node assessment can be technically challenging.
Nodal staging	Yang et al., 2019 (25)	Prospective study; *n* = 116	IVIM assessment of regional lymph nodes	Interobserver agreement for IVIM-MRI parameters between two readers was excellent (ICCs >0.75) IVIM parameters showed potential for non-invasive assessment of lymph-node microstructure and microcirculation.	Supports nodal evaluation, but reproducibility and standardization remain important limitations.
Nodal staging using primary tumor histogram	Zhao et al., 2021 (52)	Prospective; *n* = 95	Histogram models from ADC, D, D*, and f maps	Combined histogram model achieved high diagnostic performance for nodal staging, with AUC = 0.959.	Promising for nodal staging, especially when conventional MRI criteria are limited.
Nodal staging in preoperatively node-negative disease	Zhao et al., 2021 (53)	Prospective single-center; *n* = 74	Multiple DWI models including IVIM and DKI	D* and distributed diffusion coefficient (DDC) were independent predictors of nodal involvement. Their combination achieved AUC = 0.872. D*, DDC, and MK (mean kurtosis) were significantly different between pN0 and pN1–2 groups (all *P* < 0.001)	Multiparametric diffusion models may improve detection of occult nodal metastasis.
APT/IVIM histogram and pathological factors	Kan et al., 2025 (43)	Prospective; *n* = 42	APT imaging and IVIM-DWI histogram	D and D* histogram parameters predicted lymph node metastasis and vascular invasion. f showed no significant value for the evaluated pathological factors.	Suggests histogram analysis may improve prediction of adverse pathology, but sample size was limited.

IVIM has demonstrated incremental diagnostic value over conventional MRI in selected clinical scenarios; however, its added benefit remains inconsistent across studies (26, 27). A prospective study of 46 patients reported significantly higher f and D* values in lymphovascular invasion (LVI)-positive tumors than in LVI-negative tumors, although their discriminatory performance was limited (28). However, studies evaluating conventional IVIM quantitative parameters have demonstrated relatively limited diagnostic performance for identifying lymphovascular invasion. Although f and D* were significantly higher in LVI-positive tumors, their discriminatory performance was limited (AUCs 0.629 and 0.647, respectively), and the corresponding ROC analyses were not statistically significant (28). Moreover, IVIM can be used to distinguish normal rectal tissue from tumor parenchyma. Moreover, Lu et al. (29) found that D, D*, and f significantly differentiated rectal tumor parenchyma from normal rectal wall, but D and D* did not clarify differences among carcinomas.

### 4.1 IVIM for primary tumor characterization

Several studies have been conducted on tumour characterization in rectal cancer using IVIM (8, 22, 30, 31). Current evidence suggests that IVIM can provide complementary biological information beyond conventional anatomical MRI in rectal cancer. Across the included studies, the true diffusion coefficient D appears to be the most biologically interpretable IVIM parameter because it reflects tissue cellularity and microstructural restriction. Lower D values have generally been associated with more aggressive tumor features, including poorer differentiation, higher tumor stage, increased cellular density, and adverse pathological characteristics. This relationship is biologically plausible: increased tumor cellularity reduces the extracellular space available for water movement, thereby increasing diffusion restriction and resulting in lower *D* values. Consistent with this, greater architectural complexity and densely packed malignant cells—particularly in poorly differentiated or more aggressive tumors—may further restrict water diffusion (10, 22, 32). This pattern is supported by studies by Surov et al. (22), Geng et al. (2020) (8), Yuan et al. (32), Zhou et al. (10), and Zhou et al. (33). Additional evidence from Li et al. (34) showed lower D values in high-grade tumors and in tumors with pathological EMVI, further supporting an association between adverse pathological features and restricted true diffusion; however, IVIM parameters did not consistently differentiate several other clinicopathological subgroups. However, these associations are not uniform across all studies. For example, Sun et al. (35) found that IVIM parameters did not reliably differentiate early-stage pT1–2 tumors from pT3a tumors, suggesting that IVIM performance may depend on tumor stage distribution, sample size, segmentation approach, and acquisition protocol.

In contrast, the perfusion-related parameters f and D* have shown more variable clinical performance. Some studies reported associations between f or D* and lymphovascular invasion, tumor vascularity, differentiation grade, or risk stratification, suggesting that these parameters may reflect microcirculatory or angiogenic features of rectal tumors. This was observed in studies by Sun et al. (36), Meyer et al. (37), Li et al. (31), Chai et al. (38), and Zhou et al. (10). For example, the relationship between histological differentiation and f remains inconsistent across studies. Lu et al. reported (29) lower f values in poorly differentiated tumors than in well- and moderately differentiated tumors, potentially reflecting rapid tumor growth accompanied by relatively insufficient or immature microvascular development. Similarly, Sun et al. (36) observed lower perfusion-related IVIM parameters in poorly differentiated tumors. In contrast, Li et al. (31) reported higher f values in poorly differentiated tumors, suggesting that abnormal neovascularization and increased vascular permeability may also influence the perfusion fraction in more aggressive tumors. These conflicting findings highlight the complex relationship between microcirculation and tumor differentiation and suggest that f is influenced by both methodological factors and biological heterogeneity.

Nevertheless, other studies reported weak or non-significant correlations between perfusion-related IVIM parameters and histopathological markers, including Bakke et al. (30), Yuan et al. (32), and Sun et al. (35). This inconsistency likely reflects the known instability of perfusion-related IVIM metrics, which are highly sensitive to low b-value selection, SNR, motion, fitting algorithms, scanner platform, and region-of-interest methodology. Therefore, while f and D* may provide biologically relevant information in selected settings, they should currently be interpreted as exploratory rather than robust clinical biomarkers.

Several studies also suggest that IVIM-derived parameters may be useful for predicting molecular or adverse pathological features. Xu et al. (39) reported that IVIM parameters differed according to KRAS mutation status, with lower D and higher D* values in KRAS-mutant tumors. Yan et al. also reported differences in f according to mismatch-repair protein status, although its modest discriminatory performance (AUC 0.695) suggests that this application remains exploratory (40). Yang et al. (41) showed that histogram-derived D metrics were associated with tumor budding and prognostic stratification, while Fu et al. (42) similarly reported that a model incorporating IVIM and clinical-anatomical features improved identification of high-grade tumor budding. Kan et al. (43) further suggested that IVIM histogram parameters may help predict lymph node metastasis and vascular invasion. Whole-tumor IVIM histogram analysis has also shown potential for assessing perineural invasion. He et al. (44) identified histogram-derived IVIM features associated with PNI status, while Yang et al. (45) reported that combined IVIM histogram and clinical models improved PNI prediction and were also associated with disease-free survival.

IVIM changes have also been demonstrated in the mesorectal microenvironment. Lu et al. (46) reported differences in mesorectal D and f according to MRI-detected circumferential resection margin and extramural vascular invasion status, suggesting that IVIM may capture microstructural changes associated with local tumor spread; however, the clinical significance of these findings requires further validation. Another study reported that D*, f, and other diffusion-model parameters differed according to pathological EMVI in patients with negative conventional MRI findings, while a combined model incorporating diffusion parameters and tumor location provided better diagnostic performance than individual parameters (47). However, these applications remain preliminary because most studies are single-center, use heterogeneous MRI protocols, and lack external validation.

Overall, IVIM-derived parameters appear most useful when interpreted within a multiparametric imaging framework rather than as isolated biomarkers. Combining IVIM with diffusion kurtosis imaging, dynamic contrast-enhanced MRI, histogram analysis, or radiomics has generally improved diagnostic performance compared with individual IVIM parameters alone (8, 38, 48, 49). This improvement likely reflects the complementary biological information provided by diffusion, perfusion, and measures of intratumoral heterogeneity. However, improved diagnostic performance within individual study cohorts does not necessarily indicate clinical generalizability, particularly because most available studies were conducted in relatively small, single-center populations using heterogeneous acquisition and post-processing methods. Therefore, standardized acquisition, reproducible post-processing, and independent external validation are required before these multiparametric approaches can be translated into routine clinical practice.

### 4.2 Reproducibility and reliability of IVIM parameters

The reliability of IVIM-derived parameters has been examined in a limited number of studies, which is an important consideration for their potential clinical application. A central finding of the available reproducibility literature is that IVIM parameters differ substantially in measurement stability. Sun et al. (19) evaluated short-term test–retest, intraobserver, and interobserver reproducibility in 26 patients with rectal cancer. The intraobserver repeatability coefficients for ADC, D, f, and D* were 5.4%, 11.1%, 55.4%, and 40.3%, respectively, whereas the corresponding interobserver coefficients were 10.9%, 41.6%, 134.0%, and 177.6%. Test–retest repeatability coefficients were 19.1% for ADC, 24.5% for D, 126.3% for f, and 197.4% for D*, demonstrating substantially better short-term repeatability for ADC and D than for the perfusion-related parameters. Yang et al. (20) similarly reported only moderate reproducibility of perfusion-sensitive IVIM measurements, with an ICC of 0.71 (95% CI, 0.53–0.82) for f and 0.55 (95% CI, 0.32–0.72) for D*.

From a clinical perspective, these repeatability estimates are particularly relevant to longitudinal monitoring. The repeatability coefficient represents the approximate magnitude of change below which differences between repeated measurements may be attributable to measurement variability in 95% of measurement pairs. Thus, under the protocol used by Sun et al. (19), a change in D of less than approximately 25% could not confidently be distinguished from test–retest variability. In contrast, the very large repeatability coefficients for f (126.3%) and D* (197.4%) indicate that even substantial longitudinal changes in these parameters may fall within expected measurement variability. These values should not be interpreted as validated clinical response cutoffs, because repeatability is protocol-dependent; rather, they illustrate why D is currently more suitable than f or D* for longitudinal assessment. Until standardized acquisition and post-processing methods establish reproducible, externally validated thresholds, f and particularly D* should not be used as standalone parameters to guide individual-patient treatment decisions.

### 4.3 IVIM for nodal staging

Accurate identification of metastatic lymph nodes is essential for reliable staging and optimal management of rectal cancer. The evidence for IVIM-based nodal staging is promising but heterogeneous. Direct lymph-node IVIM studies have produced inconsistent findings (24, 25, 50). Yu et al. reported significantly lower D and D* values in metastatic than nonmetastatic mesorectal lymph nodes, whereas Qiu et al. (50) found higher D, f, and ADC but lower D* in metastatic nodes, despite D showing the highest diagnostic performance (AUC 0.94) in their cohort. These opposing results indicate that the direction and magnitude of IVIM changes in metastatic nodes are not uniform across studies and may depend on acquisition methodology, pathological composition, nodal size, and sampling. Long et al. (51) further showed that the diagnostic value of IVIM may vary according to lymph-node size. D differentiated metastatic from nonmetastatic nodes measuring 3–5 mm, whereas f was more informative in nodes measuring >5–7 mm. In larger nodes (>7 mm), IVIM parameters did not significantly distinguish metastatic from nonmetastatic disease. These findings suggest that lymph-node size may partly contribute to the variability reported across nodal IVIM studies.

Where lower D values have been observed in metastatic lymph nodes, this finding is biologically plausible because replacement of normal nodal architecture by densely packed malignant cells reduces extracellular space and restricts microscopic water movement. A similar mechanism may explain the lower D values reported in more aggressive primary rectal tumors, where increased cellularity and greater microstructural complexity are associated with restricted diffusion (22, 24, 25, 32). In contrast, the biological interpretation of D* is less straightforward. Because D* reflects the pseudo-diffusion component related to microvascular blood flow, changes in D* may reflect alterations in microvascular density, blood-flow velocity, and disorganized tumor-associated microcirculation. However, D* is also highly sensitive to image noise, low-b-value sampling, and fitting instability, so its biological associations should be interpreted more cautiously than those of D (19, 20, 30). Yu et al. reported that D was significantly lower in metastatic nodes compared to nonmetastatic nodes, particularly in smaller lymph nodes (25).

More recent studies have shifted toward using whole-tumor and histogram-based models derived from the primary tumor. Zhao et al. (52) reported that combined histogram models incorporating IVIM parameters achieved high diagnostic performance for nodal staging, while Zhao et al. (53) showed that D* combined with other diffusion metrics improved detection of nodal involvement in preoperatively node-negative rectal cancer. In Zhao et al. (52), researchers extracted 72 histogram features from IVIM parametric maps (ADC, D, D*, and f) of the primary tumor for each case. All parameters demonstrated good to excellent ICCs (0.8). The study found that the f map-based model demonstrated superior diagnostic efficacy compared with other single-map models, and the final combined model was developed using D* and f map-derived parameters, indicating that nodal staging of rectal cancer is more closely associated with microvascular perfusion. The combined model incorporating multiple histogram parameters showed superior diagnostic performance for predicting nodal metastasis compared to conventional MRI staging or single-parameter models. This combined model, based on several maps, reported an AUC of 0.959, along with sensitivities of 94.3%, specificities of 88.3%, and an accuracy of 90.5%. Importantly, the use of more advanced IVIM parameters does not necessarily improve nodal staging performance. Wang et al. found that IVIM and DKI did not significantly outperform conventional DWI in detecting metastatic lymph nodes, while a combined model incorporating diffusion and anatomical features showed better diagnostic performance (54).

However, the interpretation of these findings requires caution. Most nodal-staging studies are single-center and include relatively small cohorts, and the reported diagnostic performance varies according to patient selection, pathological reference standards, ROI strategy, and modeling approach. In addition, although perfusion-related parameters such as D* and f have been identified as predictors of nodal involvement in some studies, their poor reproducibility limits their reliability for individual patient-level decision-making. Therefore, IVIM-based nodal staging should currently be viewed as a promising research tool rather than a replacement for standard MRI assessment.

### 4.4 Prediction of treatment response

The IVIM technique has been most extensively investigated in the context of neoadjuvant chemoradiotherapy (nCRT) response assessment in locally advanced rectal cancer (LARC), as summarized in Table 3. Across studies, diffusion-related parameters, particularly D and ADC, show the most consistent relationship with treatment response. Responders generally demonstrate higher post-treatment diffusion values or greater percentage increases in D and ADC, reflecting reduced cellularity, necrosis, and treatment-induced microstructural changes. This pattern is supported by Lu et al. (26), Hu et al. (59), Li et al. (9), and Yang et al. (60). For example, Lu et al. (26) investigated the role of IVIM imaging in predicting pCR in a prospective cohort of 42 patients with LARC undergoing nCRT. They found no significant differences in the D* and f values (*P* = 0.464 and 0.069, respectively). In contrast, significant differences in the ADC and D values (all *P* < 0.001) were observed between pre-nCRT and post-nCRT. Moreover, the pCR group had higher preD*, pref, postD, *Δ*% (*Δ*% parametric value), ADC and *Δ*%D values than the non-pCR group (*n* = 32) (all *P* < 0.05). These findings were supported by Nougaret et al. (61) similarly found that post-nCRT D and ADC increased and were higher in good responders, whereas f and D* were neither significantly altered by treatment nor associated with response. Whole-tumor measurements also showed substantially better interobserver reproducibility than single-section ROI analysis (61).

**Table 3:** Examples of studies using IVIM for treatment-response evaluation and post-treatment assessment in rectal cancer.

Clinical application	Study/year	Study design/sample size/field	Main parameters/method/No of b-value (s/mm^2^)	Main findings	Interpretation/limitation
Treatment response after nCRT	Lu et al., 2017 (26)	Prospective; *n* = 42	IVIM before and after nCRT	ADC and D changed significantly after treatment.	Supports IVIM for response assessment, with diffusion parameters appearing more reliable than perfusion metrics.
		1.5T	**34 b-values** (0, 10, 15, 20, 25, 30, 35, 40, 45, 50, 55, 60, 65, 70, 75, 80, 85, 90, 95, 100, 105, 110, 115, 120, 125, 130, 135, 140, 145, 150, 155, 160, 800, 1,500.	IVIM parameters were able to discriminate between good and poor responders to nCRT. Perfusion-related parameters (f, D*) showed significant differences between responders and non-responders both before and after treatment.	
Treatment response and survival	Bakke et al., 2017 (62)	Prospective; *n* = 27	DWI/IVIM; tumor volume and f	High baseline f and smaller tumor volume predicted good response. Tumor volume and f were associated with progression-free survival.	Suggests f may have prognostic value, but small sample size limits generalizability.
		1.5T	**3b-values** (0, 300, 900)		
Treatment response/lymph-node status after nCRT	Li et al., 2022 (9)	Prospective two-center; *n* = 79	IVIM with different ROI strategies	Whole-volume ROI-derived percentage change in D predicted lymph node status, non-pCR, and poor response with AUCs of 0.810, 0.851, and 0.903, respectively.	Strong evidence supporting whole-volume D-based assessment after nCRT.
		1.5T	**11b-values** (0, 20, 50, 100, 150, 200, 400, 600, 800, 1,000, 1,200)		
Treatment response after nCRT	Hu et al., 2021 (59)	Retrospective; *n* = 50	IVIM-DWI before and after nCRT	ADC and D increased after treatment. Percentage changes in ADC and D correlated with response, and %D outperformed %ADC for pCR prediction.	Supports D as a useful response biomarker, but retrospective design limits strength.
		3T	**11b-values** (0, 10, 50, 100, 150, 200, 400, 600, 1,000, 1,500, 2,000)		
Tumor regression grade/post-treatment T stage	Yang et al., 2021 (60)	Prospective single-center; *n* = 42	IVIM and DKI after nCRT	Post-nCRT MD showed the highest diagnostic performance for pCR. Post-nCRT ADC, D*, f, and MD were higher in pCR and low ypT-stage groups.	Suggests combined IVIM-DKI may improve post-treatment response assessment.
		3T	**9 b-values** (0, 25, 50, 75, 100, 125, 150, 200, 800)		
Complete response prediction	Zhang et al., 2025 (48)	Prospective; *n* = 59	Histogram analysis of IVIM and DKI	Combined IVIM-DKI histogram model improved prediction of complete response compared with conventional MRI.	Highlights added value of histogram-based multiparametric analysis.
		3T	**9 b-values** (0, 20,40,80, 160,400,600,800, 1,000)		
Response prediction using histogram and clinical model	Yang et al., 2025 (49)	Retrospective; *n* = 112	IVIM histogram parameters plus clinical characteristics	Combined model using IVIM histogram parameters and clinical features achieved high performance for predicting pCR (AUCs 0.916)	Promising, but requires external validation before clinical use.
		3T	**8 b-values** (0, 50, 80, 100, 200, 500, 800, 1,000)		

The predictive role of perfusion-related parameters is less consistent. Some studies reported that higher baseline f or D* values were associated with better response, possibly reflecting better tumor oxygenation and treatment sensitivity. Although Bakke et al. (62) reported an association between higher baseline f and favorable response, and Lu et al. (26) and Hu et al. (59) identified associations between selected perfusion-related parameters and pathological response, other studies found diffusion-related changes, particularly *Δ*D and *Δ*ADC, to be more robust predictors (9, 59). These findings indicate that associations between f or D* and treatment response have not been consistently reproduced across cohorts. Moreover, not all studies have demonstrated an incremental advantage for IVIM over alternative diffusion models. Zhu et al. (63) reported that ADC and the stretched-exponential *α* parameter were more useful for assessing pCR than conventional IVIM-derived parameters. Similarly, Liang et al. (64) found that only Dslow among the IVIM parameters differed significantly between pCR and non-pCR groups, while overall IVIM performance was lower than that of conventional ADC and the stretched-exponential model.

Importantly, studies using whole-volume ROI analysis, histogram features, or combined IVIM-DKI models generally reported better predictive performance than those relying on mean IVIM values alone. Li et al. (9) showed that whole-volume ROI-derived percentage change in D was an independent predictor of lymph node status, non-pathological complete response, and poor tumor response. Zhang et al. (48) demonstrated that histogram analysis combining IVIM and DKI improved the prediction of complete response to neoadjuvant therapy. Similarly, Yang et al. (49) showed that combining IVIM histogram parameters with clinical characteristics improved response prediction. These findings indicate that treatment response is not fully captured by average tumor diffusion or perfusion values. Instead, intratumoral heterogeneity and multiparametric modeling may better reflect the complex biological changes induced by neoadjuvant therapy. Nevertheless, most predictive models remain insufficiently validated, and their clinical use requires confirmation in larger multicenter cohorts. Beyond initial treatment-response assessment, IVIM may also have a role in post-treatment surveillance. Fusco et al. (65) reported that a multiparametric model combining conventional DWI, IVIM, and DKI features improved differentiation of local recurrence from post-treatment scar or fibrosis, although validation in larger independent cohorts is required.

## 5 IVIM radiomics

Radiomics and histogram-based analysis are important advances because they move beyond simple mean IVIM values and enable assessment of intratumoral heterogeneity. Across published studies, combined models that integrate IVIM parameters with radiomics features, texture analysis, clinical variables, or conventional MRI have generally outperformed individual IVIM parameters in predicting treatment response, lymph node metastasis, lymphovascular invasion, extramural vascular invasion, tumor differentiation, and molecular features (35, 49, 66–70). Su et al. similarly showed that combining clinicopathological characteristics, f, and texture features improved prediction of nCRT resistance compared with individual parameters, although the retrospective single-center design and internal validation limit its generalizability (71). Moreover, Sun et al. (35) evaluated whether texture features combined with functional MRI parameters could differentiate early-stage (pT1–2) from locally advanced (pT3a) rectal cancer. Although IVIM parameters alone did not show significant differences across tumor stages, the study identified several texture features and additional MRI parameters, including ADC and dynamic contrast-enhanced MRI metrics, that improved classification performance. A combined model incorporating texture features, ADC, and DCE-MRI parameters achieved the highest diagnostic accuracy, with an AUC of 0.833. However, the current radiomics evidence remains preliminary. Most studies are retrospective, single-center, and based on relatively small datasets. Many models lack external validation and are prone to overfitting, especially when many imaging features are extracted from limited sample sizes. Differences in segmentation, feature extraction, image preprocessing, and scanner protocols further limit reproducibility. Therefore, IVIM-based radiomics is promising but should not yet be considered ready for clinical implementation without multicenter validation and standardized analytic pipelines. As shown in Table 4, representative studies applied IVIM-derived parameters together with radiomics.

**Table 4:** IVIM-derived histogram, texture, and radiomics studies in rectal cancer.

Clinical endpoint	Study (Year)	Study type/Sample size	IVIM/radiomics approach	Best performance	Key message	Major limitation
Pathological complete response after nCRT	Liu et al. (66) (2019)	Prospective 41	IVIM-DWI with GLCM texture analysis	AUC = 0.986	Texture features outperformed conventional IVIM metrics for pCR prediction.	Very small cohort with only 9 pCR cases; risk of overfitting.
Aquaporin-1 (AQP1) expression prediction	Chen et al. (72) (2022)	Prospective 110	Multiparametric MRI with IVIM-based radiomics nomogram	AUC = 0.636	IVIM diffusion parameter D correlated with AQP1 expression.	Limited diagnostic performance of IVIM alone.
Lymphovascular invasion (LVI) detection	Wong et al. (67) (2024)	Retrospective 83	Radiomics from T2WI, DWI, and IVIM maps	Validation AUC = 0.920	Combining IVIM with radiomics improved LVI detection.	Small validation cohort.
Early T-stage differentiation (pT1–2 vs. pT3a)	Sun et al. (35) (2022)	Retrospective 82	IVIM, ADC, DCE-MRI, and texture features	AUC = 0.833	Multiparametric models outperformed IVIM alone.	Retrospective single-center study.
Non-enlarged lymph node metastasis prediction	Jia et al. (68) (2022)	Retrospective 126	IVIM-DWI with radiomics nomogram	Validation AUC = 0.942	Combined IVIM-radiomics model improved occult nodal metastasis detection.	Lack of external validation.
Extramural vascular invasion (EMVI) prediction	Gao et al. (69) (2022)	Retrospective 96	IVIM parameters with texture analysis	AUC = 0.821	D* combined with texture features improved EMVI prediction.	Moderate specificity (61.5%).
Histological grade prediction	Gao et al. (70) (2024)	Retrospective 71	IVIM texture analysis	AUC = 0.860	Combined IVIM-texture features improved tumor grading.	No external validation.
Treatment response/pCR prediction	Yang et al. (49) (2025)	Retrospective 112	IVIM histogram analysis with clinical variables	AUC = 0.916	Histogram features enhanced prediction of treatment response.	Retrospective design.

## 6 Discussion

IVIM-DWI is a non-invasive MRI technique that provides simultaneous information about tissue diffusion and microvascular perfusion without the need for contrast agents. The current evidence suggests that the clinical potential of IVIM MRI in rectal cancer differs substantially across its derived parameters and applications. Among conventional IVIM metrics, the true diffusion coefficient (D) appears to have the strongest combination of biological interpretability, reproducibility, and consistency across studies. Lower D values have generally been associated with increased tumor cellularity and adverse pathological characteristics, supporting the concept that densely packed malignant cells, reduced extracellular space, and greater microstructural complexity restrict water diffusion (8, 10, 22, 32, 33) In contrast, the perfusion-related parameters f and D* have shown more variable associations with tumor differentiation, microvascularity, and pathological risk factors (10, 30, 31, 36, 37). These findings suggest that D is currently the most robust conventional IVIM-derived parameter. In contrast, f and, in particular, D* should still be regarded as exploratory biomarkers rather than reliable standalone measures for clinical decision-making.

The biological interpretation of these differences is important when evaluating the apparent diagnostic value of IVIM. D primarily reflects the movement of water molecules within the extravascular tissue compartment and is therefore influenced by cellular density, extracellular space, membrane integrity, and tissue architecture. This provides a plausible explanation for the lower D values reported in more aggressive primary tumors and metastatic lymph nodes (24, 25). Similarly, increases in D following nCRT are biologically consistent with treatment-induced cell death, loss of membrane integrity, necrosis, and expansion of the extracellular space, all of which permit greater water mobility (9, 26). The interpretation of f and D* is more complex. Although these parameters are intended to reflect microvascular perfusion and pseudo-diffusion, respectively, their associations with other perfusion measures and histopathological features have varied across studies (30, 31, 37). Tumor angiogenesis itself is heterogeneous, with abnormal, tortuous, and functionally inefficient microvasculature, and therefore changes in IVIM perfusion parameters may not correspond directly to microvessel density or conventional perfusion measurements. Furthermore, the lower measurement stability of f and D* makes it difficult to determine whether inconsistent findings reflect true biological differences or technical variability.

Methodological heterogeneity appears to explain the conflicting results across the IVIM literature. Parameter estimation varies with the number and distribution of b-values, particularly low-b sampling, SNR, scanner characteristics, motion, fitting algorithm, and region-of-interest methodology. These factors have a particularly important influence on f and D*, which depend heavily on the rapid signal-decay component at low b-values. Sun et al. (19) demonstrated substantially poorer test–retest repeatability for f and D* than for ADC and D, while Yang et al. (20) similarly reported lower reproducibility for perfusion-sensitive IVIM parameters. Differences in segmentation strategy also contribute to variability. Li et al. demonstrated that whole-volume and other ROI approaches can influence the predictive performance of IVIM measurements (9). Therefore, apparently conflicting associations between IVIM parameters and pathological or treatment-related outcomes should not necessarily be interpreted as contradictory tumor biology. Instead, at least part of this inconsistency likely reflects differences in acquisition, parameter fitting, tumor segmentation, and study design. This methodological dependence represents one of the most important barriers to comparing quantitative thresholds across studies and to translating IVIM into routine clinical practice.

From a clinical perspective, IVIM currently appears more promising as an adjunct to established rectal MRI than as a standalone decision-making technique. For primary tumor characterization, D may provide complementary information regarding cellularity and aggressive pathological features; however, substantial overlap in parameter values between pathological groups limits the clinical applicability of single quantitative thresholds (8, 10, 32, 33, 35). For nodal staging, direct assessment of mesorectal lymph nodes has demonstrated differences in D and D* between metastatic and nonmetastatic nodes (24, 25), while more recent whole-tumor histogram approaches have reported encouraging diagnostic performance for predicting nodal involvement (52, 53). These findings are clinically relevant because conventional nodal assessment based on size and morphology remains imperfect. Nevertheless, the available evidence is insufficient to support replacement of conventional MRI assessment by IVIM. Most nodal studies have been conducted in relatively limited cohorts, and differences in patient selection, reference standards, segmentation strategies, and modeling approaches restrict generalizability. In addition, some of the strongest predictive models incorporate f or D*, whose limited repeatability raises concern regarding their reliability at the individual-patient level.

The evidence for treatment-response assessment appears comparatively more consistent for diffusion-related parameters. Across several studies, post-treatment increases or percentage changes in D and ADC were associated with pathological response following nCRT (9, 26). These observations are biologically plausible because successful treatment reduces viable tumor cellularity and alters tissue microstructure, thereby increasing water diffusion. In contrast, the predictive value of f and D* remains less consistent. Higher baseline perfusion fraction and changes in perfusion-related IVIM parameters have been associated with favorable treatment response in some cohorts (26, 62); however, these relationships have not been reproduced uniformly. Differences in treatment regimens, timing of post-treatment MRI, b-value distribution, ROI methodology, and fitting techniques may contribute to these discrepancies, in addition to the intrinsic measurement instability of f and D*. Therefore, D currently appears more promising than perfusion-related IVIM parameters for response assessment. However, even for D, the available evidence does not support a universally applicable threshold for identifying pathological complete response or altering established treatment pathways. Before IVIM can contribute to decisions such as organ-preservation strategies or modification of surgical management, its incremental value over conventional MRI and standard DWI must be demonstrated prospectively.

The increasing use of whole-tumor histogram analysis, multiparametric MRI, and radiomics represents an important development because these approaches move beyond reliance on a single mean IVIM value. Conventional mean D, f, and D* measurements may obscure spatial differences in cellularity, necrosis, fibrosis, vascularity, and treatment-related changes within the tumor. Histogram-based analysis preserves information about the distribution of voxel values, while radiomics can quantify more complex intensity and texture patterns that may better reflect intratumoral heterogeneity. This provides a plausible explanation for the improved diagnostic or predictive performance reported when IVIM-derived information is combined with DKI, texture features, conventional MRI findings, or clinical characteristics (35, 48, 49, 67–70). However, higher model performance should not automatically be interpreted as superior clinical utility. Most radiomics and histogram studies remain retrospective or single-center, and several lack independent external validation (35, 49, 67–70). Furthermore, extracting a large number of imaging features from relatively small datasets increases the risk of model overfitting and optimistic estimates of diagnostic performance. Consequently, future studies should focus not only on achieving higher AUC values but also on demonstrating reproducibility, calibration, external validity, and clinically meaningful improvement over simpler imaging models.

A key conclusion from the current evidence is that future development of IVIM in rectal cancer should shift from identifying additional statistically significant associations toward demonstrating measurement robustness and added clinical value. Multicenter prospective studies using harmonized acquisition protocols are needed, with particular attention to the number and distribution of b-values, especially in the low-b-value range. Fitting algorithms, motion-correction approaches, image quality criteria, and segmentation strategies should be clearly reported and, where possible, standardized. Whole-tumor analysis may provide a more representative assessment of heterogeneous rectal tumors than single-slice or small-ROI methods, but its reproducibility should also be formally evaluated. Given the documented measurement variability of perfusion-related IVIM parameters, particularly f and D*, future studies proposing quantitative thresholds should incorporate test–retest assessment to establish measurement repeatability (19, 20). Predictive models should also undergo independent external validation and should be compared with established clinical and MRI predictors to determine whether IVIM provides meaningful incremental information rather than statistical association alone.

This review also highlights limitations in the current evidence base. Many available studies are retrospective, single-center investigations with relatively small sample sizes, and acquisition and post-processing methods vary considerably. External validation is uncommon, particularly for histogram-based and radiomics models, which limits confidence in their generalizability. In addition, the present review was designed as a structured narrative review rather than a systematic review or meta-analysis. These factors should be considered when interpreting the conclusions. Nevertheless, the available literature consistently identifies methodological standardization and reproducibility as central challenges to the clinical translation of IVIM.

## Conclusion

IVIM provides biologically relevant quantitative information regarding diffusion and microvascular perfusion in rectal cancer, but the maturity of the evidence differs across parameters. D currently appears to be the most reproducible and biologically interpretable IVIM metric and shows the most consistent potential for tumor characterization and treatment-response assessment. In contrast, f and D* remain constrained by substantial measurement variability, protocol dependence, and inconsistent biological associations. Histogram analysis, multiparametric imaging, and radiomics may enhance the information obtained from IVIM by capturing tumor heterogeneity and complementary imaging characteristics, but their apparent advantages require confirmation in externally validated multicenter studies. At present, IVIM should therefore be considered a promising complementary quantitative technique rather than a replacement for established rectal MRI. Standardized acquisition and post-processing, reproducibility assessment, and prospective demonstration of incremental clinical value will be essential before IVIM-derived biomarkers can be incorporated into routine patient management.
